# Pandemic impacts and experiences after disaster in Australia: qualitative study of compound impacts following the Black Summer bushfires

**DOI:** 10.1192/bjo.2023.648

**Published:** 2024-02-02

**Authors:** S. Cowlishaw, C. O'Dwyer, C. Bowd, N. Sadler, M. O'Donnell, D. Forbes, A. Howard

**Affiliations:** Phoenix Australia – Centre for Posttraumatic Mental Health, Department of Psychiatry, The University of Melbourne, Australia

**Keywords:** Pandemic, disaster, recovery, trauma, community

## Abstract

**Background:**

The first cases of the COVID-19 pandemic in Australia were recorded in January 2020, which was during the ‘Black Summer’ bushfires of 2019–20 and prior to additional disasters in some regions. Few studies have considered the compound impact of disasters and the pandemic.

**Aims:**

To improve understanding of the impact on mental health and well-being of the pandemic in disaster-affected communities.

**Method:**

We conducted semi-structured interviews (*n* = 18) with community members and online focus groups (*n* = 31) with help providers from three regions of rural Australia affected by bushfires and the pandemic.

**Results:**

Six themes were produced: (a) ‘Pulling together, pulling apart’, describing experiences after bushfires and prior to impacts of the pandemic; (b) ‘Disruption of the ‘normal response’, encompassing changes to post-disaster recovery processes attributed to the pandemic; (c) ‘Escalating tensions and division in the community’, describing impacts on relationships; (d) ‘Everywhere you turn you get a slap in the face’, acknowledging impacts of bureaucratic ‘red tape’; (e) ‘There are layers of trauma’, highlighting intersecting traumas and pre-existing vulnerabilities; and (f) ‘Where does the help come from when we can't do it?’, encompassing difficulties accessing services and impacts on the helping workforce.

**Conclusions:**

This study furthers our understanding of compound disasters and situates pandemic impacts in relation to processes of adjustment and recovery from bushfires. It highlights the need for long-term approaches to resilience and recovery, investment in social infrastructure, multi-component approaches to workforce issues, and strategies to increase mental health support and pathways across services.

Across 2019–20, Australia was affected by a global pandemic and a series of climate-induced disasters that produced complex recovery environments for individuals and communities. By way of illustration, the first confirmed case of COVID-19 in Australia was recorded in January 2020, which was during the unprecedented ‘Black Summer’ bushfires of 2019–20. These fires were active over a 9 month period (from July 2019 to March 2020) and would eventually burn more than 24 million hectares, predominately across eastern and southern Australia, directly causing 33 deaths and more than 400 additional premature deaths attributed to smoke inhalation.^1^ By March 2020, COVID-19 was declared a pandemic by the World Health Organization, and public health measures were put in place by Australian state and federal governments to protect life and contain the virus. Although public health measures varied over time and according to location (with particularly severe restrictions across metropolitan areas), there were many restrictions active in rural areas that were affected by bushfires. These included restrictions on social gatherings, limited travel to regional communities and enforced closures of non-essential businesses.^2^

The intersection of environmental, human and social consequences of disasters and the pandemic have supported expectations of major impacts on the mental health and well-being of many Australians. These have been substantiated by studies conducted following the arrival of the pandemic in Australia, which have indicated overall declines in mental health relative to pre-pandemic levels.^3^ However, relevant studies also suggest that the magnitude of declines differed across population subgroups (for example, impacts appear to be greater among individuals with pre-existing mental health problems) and also varied across stages of the pandemic.^3^ For example, longitudinal studies^4^ have suggested that distress increased at the end of April 2020, when public health measures were at their highest levels and then declined subsequently as restrictions eased in many regions. Such findings are broadly aligned with studies conducted after earlier disasters, which also outline trajectories of mental health impacts that must be discerned over time^5–7^ and may be influenced by stressors that follow the onset of such events, for example, being associated with difficulties re-establishing businesses and navigating insurance or assistance programmes.^8^

There is a discrete body of literature on the mental health and well-being impacts of compound disasters, which refer to ‘combinations of simultaneous or successive extreme hazard events’.^9^ Although nascent, this literature highlights cumulative impacts of such events and suggests the need for tailored approaches to conceptualising, supporting and sustaining recovery in the context of different types and sequences of disasters.^9^ However, there is a pressing need to better understand the impacts of compound events and their implications for mental health and well-being, given the projected increases in disasters due to climate change.^10^ Notwithstanding the unique features of major bushfires and a public health emergency, few studies have yet considered specific impacts on mental health and well-being of the Black Summer bushfires and COVID-19 pandemic. Available studies have mainly comprised cross-sectional surveys of adolescents^11^ and adults,^12^ teachers^13^ and older adults,^14^ with one study using a mood-monitoring application that tracked changes over time.^15^ The latter suggested that symptoms of depression increased during the bushfires and remained elevated subsequently during pandemic restrictions, whereas experiences of anxiety and reduced social connectedness were observed during the pandemic (relative to when bushfires were active).^15^ Cross-sectional surveys also indicate that bushfire and pandemic impacts are both associated with reduced mental health^11^ and well-being^13^ (although links with pandemic stressors may be attenuated when controlling for factors such as financial difficulties),^12^ as well as greater social isolation.^14^ By contrast, relevant studies have found no evidence of interactions between pandemic and bushfire impacts, although complex relationships have been observed with adverse childhood experiences (suggesting compounding effects of disasters and earlier trauma histories).^11^

Fewer studies still have used qualitative designs to generate deeper understandings of the impacts of bushfires and the pandemic, and these have focused narrowly on experiences of loneliness and social isolation in the context of pandemic restrictions^16^ and experiences of persons with specific health conditions such as multiple sclerosis.^17^ This relative dearth of qualitative evidence has been identified as a gap in research conducted after the pandemic.^3^

## Current study

The broad goal of the current study was to improve understanding of the compound impacts on mental health and well-being of the pandemic in bushfire-affected communities and implications for improving responses to compound disasters. This was addressed using interviews with community members and focus groups with help providers in communities that were affected by both bushfires and the pandemic. These were guided by research questions including the following.

- How did community members experience and perceive impacts on mental health and well-being of the pandemic and additional disasters?
- How did they perceive their mental health needs, challenges and facilitators to recovery?
- How did help providers describe their experiences of service delivery and challenges to or facilitators of recovery?

## Method

### Participants and procedure

Recruitment materials were shaped by an advisory group comprising individuals who had experienced or supported communities affected by COVID-19 and other disasters and was established for this research to provide advice regarding recruitment, protocol development and finding implications. These were targeted at regional areas of three Australian states (New South Wales (NSW), Victoria and South Australia) that were heavily affected by the Black Summer bushfires and COVID-19 pandemic. Several regions were also affected by subsequent disasters across 2021 and 2022, such as major flood events (NSW) and severe storms and floods (Victoria). Potential participants were invited to take part via geo-targeted social media posts and emails distributed to a database of contacts from disaster-affected communities who had previously engaged with the researchers, as well as flyers distributed to local government councils and other networks. Materials invited participants to take part in semi-structured interviews or focus groups according to whether they were: (a) community members (who were mainly invited to interviews) or (b) help providers (who were mainly invited to focus groups). The definition of a ‘help provider’ was broad, and although participants were predominantly employees of mental health and well-being services, there were several who undertook volunteering roles with community organisations. This broad distinction between participant types was notwithstanding that many individuals in rural communities hold dual roles, whereby help providers are also community members (and in some instances people who were invited for focus groups ended up participating in interviews and *vice versa*).

Recruitment activities produced *n* = 18 semi-structured interviews that were conducted via telephone in April 2022. Interviews ranged from 35 to 65 min (mean = 50) and were audio-recorded and transcribed verbatim. There were *k* = 5 online focus groups conducted using videoconferencing software between April and May 2022. Focus groups ranged from 25 to 90 min in duration (mean = 72) and were audio-recorded and transcribed verbatim. The number of participants in each group ranged from four to eight, with *n* = 31 participants taking part in focus groups. Consent to participate was reaffirmed with all participants at the start of each interview or focus group, either verbally or via a written consent form.

Interviews and focus groups used a semi-structured interview guide and focus group protocol that were developed for this project. The interview guide included questions on personal experiences of disasters and the pandemic, impact on mental health and well-being, and facilitators or barriers to coping and help-seeking. The focus group protocol included questions regarding perceptions of impacts of the pandemic and disasters on the mental health and well-being of communities, observations of coping and help-seeking behaviours, and the role and challenges of help providers.

The authors assert that all procedures contributing to this work comply with the ethical standards of the relevant national and institutional committees on human experimentation and with the Helsinki Declaration of 1975, as revised in 2008. All procedures involving human subjects/patients were approved by the University of Melbourne Human Research Ethics Committee, approval number: 2022-23083-25644-3.

### Data analyses

Analyses adopted a social constructivist framework using principles of reflexive thematic analysis.^18,19^ Inductive methods were used to code data and involved moving from descriptive to interpretative codes, and then to overarching themes. Relevant statements were coded with ample context to avoid data fragmentation and de-contextualisation.^20^ The coding was initially undertaken by two researchers (C.O. and C.B.) who were focused on interviews and focus groups, respectively. In both instances, coding was conducted iteratively with co-researchers, and a selection of transcripts and quotes were reviewed by co-authors under each theme.^21^ Once the themes for community members and help providers had been developed, two primary researchers (C.O. and C.B.) reviewed and synthesised the overlapping themes to create a cohesive narrative that encompassed the findings derived from interviews and focus groups.

## Results

Interviewees and focus group participants reported basic sociodemographic information. Most interviewees (78%) identified as women; whereas 33% reported being currently employed on a casual basis, 28% were retired, 22% were employed full-time and 11% were unemployed. Interview participants ranged in age from 35 to more than 65 years old. Half of interviewees were located in regional NSW, 44% were from regional Victoria and 6% were from regional South Australia. Almost all focus group participants (94%) also identified as women. Focus groups were organised broadly based on location, with three groups involving participants from Gippsland and north-east regions in Victoria and two involving participants from the mid-coast and south coast regions of NSW. Participants worked in healthcare (including mental health) organisations (29%), in education roles (29%), for community organisations (23%) or for local, state or federal government (19%). The majority (61%) of focus group participants did not provide their employment status; however, 16% indicated that they were employed in a full-time capacity, 13% part time and 10% in casual roles. The small number of participants from South Australia and the small number of men that took part reflected the varying degrees of success of our recruitment activities and were not intentional features of the study design.

### Qualitative findings

Six broad themes were derived from the interviews and focus groups. These are summarised in Table 1 and described in detail below.

**Table 1:** Summary of themes derived from interviews and focus groups

Theme	Summary
Pulling together, pulling apart	Encompassed experiences reported during the initial period following the bushfires and prior to the impacts of the pandemic. These included an initial sense of coming together in the community, followed by a growing sense of strain (including emerging mental health problems) and interpersonal tension.
Disruption of the ‘normal response’	Encompassed changes to typical post-disaster recovery processes that were attributed to the pandemic. These referenced impacts of public health restrictions and social distancing on community engagement and sense of stability during lockdowns. This theme also included descriptions of hopelessness and powerlessness that emerged in response to the pandemic and were compounded by subsequent disasters and the ongoing pandemic environment.
Escalating tension and community divisions	Encompassed increasing impacts of disasters and the pandemic on relationships. These referenced tensions that were attributed to contrasting attitudes to COVID-19 vaccinations, difficulties reconnecting with social groups following restrictions, and a broader sense of division and alienation from other communities that were perceived as having problems with COVID-19 or were the reason for restrictions (particularly metropolitan areas). This theme also included descriptions of changes in feelings of connectedness as pandemic restrictions eased, along with more inclusive coping strategies that were used over time.
Everywhere you turn you get a slap in the face	Encompassed impacts of bureaucratic ‘red tape’, which were pre-existing but had worsened during the pandemic. These referenced reactions to recounting experiences many times across disconnected services and struggles to access inflexible support schemes. Difficulties also occurred in the context of housing shortages, including pandemic-related and other obstacles to rebuilding properties that had been damaged in bushfires. These administrative demands were also viewed as contributing to burdens on help providers.
There are layers of trauma	Encompassed impacts of additional intersecting traumas and pre-existing mental health difficulties, which were exacerbated by disasters and then pandemic restrictions. These also reference gender-based experiences of family violence, and homelessness, which had been exacerbated by disasters and the pandemic.
Where does the help come from if we can't provide it?	Encompassed difficulties accessing services in rural areas and impacts on the helping workforce. Access difficulties referenced limited numbers of providers in rural areas (particularly those who were trauma-informed) and increased demand during the pandemic. Chronic high demand for services was described as falling on the same group of help providers, with impacts that included exhaustion, burnout and vicarious trauma.

#### Theme 1: pulling together, pulling apart

The first theme encompassed initial experiences after the Black Summer bushfires and prior to impacts of the pandemic. In this period, many participants described early experiences of coming together to commence the recovery process through sharing practical and emotional support.

‘*After the bushfire, the first three months people were helping each other, trying to find solutions, lots of get togethers. It was actually a really strong time for the community.’ (Participant from focus group 2)*

Many also reflected on emerging impacts of the bushfires on their mental health and referenced experiences of shock, anxiety, sadness, anger, ‘brain fog’, hypervigilance, tearfulness, sleep problems, difficulties concentrating and low motivation. Others reported feeling overwhelmed and a prolonged sense of shock, which created difficulties engaging in initial clean-up activities, as well as application processes for grant schemes to support recovery and help services. There were also descriptions of emerging tensions among community members, which were seemingly reflections of high emotion and exhaustion, and associated tendencies to lash out and blame others.

‘*…once the buzz had worn off after the fires everyone just really went to their corners and the engagement within the community got very, very low. It wasn't healthy and there was some … finger-pointing and stuff like that went on.’ (Community member 1)*

#### Theme 2: disruption of the ‘normal response’

The second theme encompassed perceived changes to typical post-disaster recovery processes that were attributed to the pandemic. Fear of contracting COVID-19 and social distancing restrictions were described as changing social behaviours and reducing community connection, which many participants felt contributed to a disruption of ‘normal responses’ post-bushfires.

‘*The end of the bushfires in February literally led straight into the COVID pandemic. The normal responses to a disaster, gathering, supporting each other, … couldn't happen because of COVID.’ (Participant from focus group 1)*

Lockdowns were described as affecting community engagement activities such as social events, local sports and volunteering, including via closures of physical places such as community halls and schools.

There were some community members and help providers who described impacts of additional disasters that occurred subsequent to the bushfires and pandemic and referenced feelings of pessimism about the future and a sense of powerlessness. One help provider elaborated on how service closures in the context of lockdowns affected feelings of instability and a growing sense of hopelessness.

‘*Our service is really meant to be … that constant safe place. We had to close a few times. I think it's a little bit difficult … that there's this place that people have begun to rely on. You never know if the rug's going to get swept out from under your feet. So, making those plans to look forward to the future, to start making connections, it's just a lot of hopelessness.’ (Participant from focus group 1)*

#### Theme 3: escalating tensions and division in the community

This theme encompassed accounts of compounding impacts of disasters and the pandemic on relationships, including increasing fractures attributed to pandemic restrictions and contrasting attitudes toward COVID-19 vaccinations, as well as division and alienation from other communities that were perceived as having high rates of infection. Interpersonal tensions and fractures were reported across all types of relationships, including intimate partners, extended families, neighbours and friendship networks.

‘*…there was a judgment up the Valley that we couldn't go up there because we'd bring COVID with us. There were some help packs given out. It was only my neighbour whom I dearly love who said, ‘What about [family names]? They've lost everything. You mean you're not going to give them a survival pack?’ Even they were like, ‘No.’ (Community member 8)*

Some participants described a broader sense of disengagement that was attributed to pandemic restrictions but persisted after restrictions eased and was reflected in difficulties reconnecting with social groups.

‘*We had a women's exercise group in a small town. By the end of the … lockdowns, we had two people turning up because people had just lost that enthusiasm. It's really only now that they're … ready to engage again. It's definitely not just something that you can pick up straight away. I think mentally it's taken them a long time to get back to that space where they do feel comfortable and want that routine again.’ (Participant from focus group 2)*

The compounding effects of multiple disasters and the pandemic on relationships were commonly identified by help providers, who suggested that interpersonal difficulties were visible indicators of poor adjustment across individual and community levels.

‘*I feel like after the multiple disasters, it's often about a year after that that hits you. If you get another one whack on top of that, that's pretty hard. This … community have been through … fires and floods and things, but still multiples. I think the fact that we are not all still coming together is a sign that we are not all well.’ (Participant from focus group 4)*

#### Theme 4: everywhere you turn you get a slap in the face

The fourth theme encompassed impacts and burdens of administrative and bureaucratic ‘red tape’ that were reported by both community members and help providers. This red tape was often described as a pre-existing issue that worsened during the pandemic, and was ‘re-traumatising’ for some individuals, and was also described as a source of anger.

‘*Nothing was connected. Every time someone rang you, you had to retell your story. Every single time if you needed to apply for a grant or you needed help … I must have told our bushfire story three times a day some days to different agencies. It was heart breaking. It was the same with COVID. If you are trying to get assistance we have to retell our story … You … shouldn't have to answer a million questions and go through that trauma every time you talk to somebody.’ (Community member 7)*

Despite the availability of financial support programmes from government, many community members and help providers described struggles accessing funds that were time-limited or tied to a specific disaster. The red tape also resulted in a sense of burdening help providers with administrative requirements that took time that could have been better spent providing care.

‘*You're sitting there for an hour … talking them through stuff just to get a bit of paper that they've got to get to feed for the cows … You end up doing that work but then other stuff's not happening as well. The job you're employed to do … is dropping off as well because you're busy doing the practical survival stuff.’ (Participant from focus group 3)*

Housing difficulties were described as additional challenges to recovering from disasters in the context of the pandemic. Participants referenced soaring rental prices, the disparity between the number of buildings lost and the (smaller) number rebuilt, and difficulties finding rental properties for existing community members, as well as those who had moved to the community to assist recovery efforts. In addition, new bushfire-related building standards, shortages of labour and tradespeople, and increases in the price of building materials all contributed to a sense of increasing financial pressures.

‘*Suddenly housing costs make it even more unaffordable for people trying to rebuild after the disaster. Just chatting on the housing crisis that's come as a result of COVID, in the community here affected by bushfires, they've been working really hard to get professionals into the community with staff at the school, locum GPs and all of that but there's nowhere for them to relocate to.’ (Participant from focus group 1)*

#### Theme 5: ‘there are layers of trauma’

This theme encompassed impacts of intersecting traumas and pre-existing vulnerabilities. Many participants described how the initial mental health impacts of the bushfires were exacerbated by pandemic restrictions, which resulted in further declines in mental health.

‘*With the traumas is the double whammy, bushfires, pandemic, and … it's bringing up all these other traumas as well, early childhood trauma, sexual abuse. That's why I mentioned complexity, because it's just – everything is coming up.’ (Community member 9)*

For some participants, there were additional intersections with gender-based experiences that complicated their situations further. For example, several help providers described observing increases in family violence, whereas some community members also spoke about their own experiences of violence. In the context of housing difficulties, homelessness was another intersecting issue for some men, women and those from the LGBTQIA+ community.

#### Theme 6: ‘where does the help come from when we can't do it?’

The final theme encompassed difficulties accessing services and supports owing to the limited availability of professional help in rural areas, the limited capability of service providers and adverse impacts on the helping workforce. Most participants, including community members and help providers, reflected on the general scarcity of professional help services in rural communities. These pre-existing issues were compounded by disasters that had led to increased need for support services and helping professionals who were trauma-informed.

‘*I think a lot of people ended up working … post bushfire recovery and had no background in crisis, clients, difficulties, trauma. I think some people were out of their depth and not supported. I really do feel … you do need to have some skills or knowledge … before you do it because it's unfair for everyone.’ (Participant from focus group 1)*

The pandemic was also described as having wide-ranging impacts on service access and provision. These included some descriptions of positive outcomes including new online services and telehealth, although difficulties accessing such services owing to internet connectivity and technological literacy in rural areas were discussed. However, in general the high demand for mental health services was described as resulting in long waitlists and negative impacts on the provider workforce.

‘*Waitlists got longer, and workers got burnt out fast. There's not enough workforce to meet the need that there currently is. You see positions for jobs in the area that constantly are listed for a year and not filled. Services have a year to two waitlists for specialised support.’ (Participant from focus group 2)*

Help providers reflected regularly on the emotional toll they experienced owing to work within disaster- and pandemic-affected communities. Descriptions of emotional exhaustion and burnout were common among participants, who commonly observed that the same professionals were providing support to communities affected by disasters and the pandemic. This sense of exhaustion among providers was acknowledged in both interviews with community members and focus groups with providers themselves and was described as placing a huge amount of strain on a small pool of helping professionals.

‘*When we first started, it was bushfires. So, that was our target … But then, with COVID coming, putting another layer on it; with the floods coming, we feel sometimes we're pulled here, we're pulled there. There's not enough of us to try and do all the things that everyone's asking us to do.’ (Participant from focus group 1)*

The continuous and high level of workload was described as affecting the mental health of providers and therefore their capacity to respond to subsequent disaster events such as the record-level floods that inundated the regional town of Lismore NSW in February 2022, affecting more than 2000 properties.^22^

^‘^*Vicarious trauma for … anyone on the front line is really important too. [Name of organisation] asked me to go to Lismore when it [major flood] happened. At the time, I've made personal excuses why I couldn't, but … I couldn't take much more trauma and I was protecting myself ….. I've seen colleagues that have been on this journey with me fall to the wayside and burn out and take its toll.’ (Participant from focus group 4)*

## Discussion

This study provides in-depth insights from community members and help providers in disaster-affected regions of Australia, regarding the compound impacts of the 2019/20 Black Summer bushfires and COVID-19 pandemic. It begins to address the general scarcity of qualitative evidence regarding the impacts of the pandemic^3^ and also contributes to an emerging body of research on the unique characteristics of compound disasters.^9^ This study also provides a unique contribution of gendered experiences of compound disasters, given that participants predominantly identified as female.

The first theme provided early accounts of post-disaster (and pre-pandemic) experiences, which referenced community members coming together after the bushfires but with growing recognition of mental health concerns and community tensions that emerged over time. Such accounts are consistent with observations made after previous bushfires that also described changing dynamics in which a shared focus on the disaster provides an initial sense of collective significance, which then gives way to community tensions that emerge over time.^23^ These changes align with processes described after other disasters that reference both the mobilisation and deterioration of social support over time^24,25^ and are also reflected in conceptual accounts of community recovery that have been proposed in relation to such single disasters.^26^ These suggest a U-shaped trajectory that often commences with a ‘honeymoon’ phase in the disaster's aftermath, reflecting a shared sense of survival and community engagement. This is followed by a downward trend characterised by growing frustration, loss of support and fragmentation, and a subsequent phase defined by the navigation of obstacles and an upward path towards reconstruction and recovery. This process is proposed to follow a timeline that often extends across a 1–3 year period, although research suggests that mental health impacts of major disasters may still be discernible 10 years afterwards.^7^

Findings from this study also provided accounts of how the pandemic had disrupted the aforementioned recovery processes and emphasised particular impacts on social connection and community engagement. The second theme identified that participants were often aware of the importance of community support following bushfires and recognised how social distancing and the closure of community services or spaces had reduced opportunities for connection. The third theme also described social impacts including interpersonal conflicts over vaccine attitudes, as well as divisions that reflected concerns about the spread of the pandemic from other communities. Such findings regarding adverse social and community impacts can be viewed in relation to broader literature indicating that although experiences of engaging support systems after disasters may be complex and sometimes negative,^27^ these networks can provide critical sources of practical and emotional support that minimise the mental health impacts of disaster-related trauma and adversity.^28^

The view of social and community connection as a recovery resource is consistent with principles of community capitals frameworks, which emphasise the importance of social capital and use this term to describe interpersonal connections and traditions of trust and reciprocity among people and groups.^29^ Broader accounts also distinguish between bonding social capital, which refers to strong ties that exist within groups of family, kin and close friends, and bridging social capital, which reflects looser ties across larger networks of people that may be less similar in terms of personal characteristics and identities.^30^ When viewed from this perspective, the current findings may suggest that a major impact of the pandemic was to reduce stocks of social capital in disaster-affected communities, including both bonding social capital (for example, resulting from social distancing and reduced opportunities to engage with close networks) and bridging social capital. The latter was inferred from descriptions of conflict and distrust across groups that were defined by emerging dimensions of identity that became salient during the pandemic, for example, reflecting attitudes towards and uptake of COVID-19 vaccines.

Community capitals frameworks may also provide a perspective on findings that highlight additional areas of compound impacts of disasters and the pandemic. For example, notions of built capital refer to physical infrastructure that may be damaged by disasters (e.g. homes, utilities) and typically comprises priority concerns for disaster recovery.^29^ Studies conducted after previous disasters have suggested that reestablishment and rebuilding activities are the most common stressors reported after disasters,^8^ and the current findings indicate that these processes were protracted by the pandemic, for example, owing to difficulties engaging builders and increasing building supply costs. Difficulties accessing housing may have been magnified by pre-existing housing shortages in some regions;^31^ these were identified as barriers to new help providers relocating to bushfire-affected communities and suggest interconnections with human capital in these regions. Human capital refers to the skills and abilities of people and communities that can be affected by disasters (for example, as people choose to move away rather than rebuild properties) and can encompass critical skill domains of healthcare and mental health. Findings of this study highlight that these stocks of human capital were already limited in rural and regional areas and had been further depleted by successive demands from disasters and the pandemic, as reflected by strong signs of exhaustion and burnout among help providers. This aligns with findings from recent surveys of mental health service providers in Australia, which have also identified escalating workforce pressures, personal impacts of disasters and the pandemic, and high levels of workforce instability.^32^

The current findings also identified impacts of administrative and bureaucratic systems on community members and help providers, including inflexible and single-disaster-specific policies for accessing financial support and requirements to recount experiences many times that were characterised as ‘re-traumatising’. This aligns with prior studies indicating that difficulties accessing financial assistance or compensation comprise major forms of secondary stressors,^33,34^ and that key features of programme design (e.g. ease of access) and ways of delivering support are likely to influence the experiences of disaster survivors.^27^ They also resonate with broader literature indicating that many systems of service provision, including health services, can have embedded practices that can unintentionally harm clients with trauma histories and must be reconfigured to be ‘trauma-informed’.^35^ The findings from this study suggest that financial assistance programmes present similar risks of introducing stressors for communities that have been exposed to disasters and the pandemic, which may have been compounded given the range of disconnected support schemes that were available and administered by different state and federal government agencies.

Finally, the study also highlighted pre-existing mental health vulnerabilities and additional forms of trauma and adversity that intersected with disaster and pandemic impacts. At the broadest level, such findings signal that there will be unique experiences of compound disasters across individuals and among groups that are already marginalised and have reduced access to personal and social resources. Prior studies of multiple disasters have identified uniquely affected marginalised groups, including those reporting low educational attainment, financial hardship and temporary housing.^9^ The current findings suggest that these may also include individuals with pre-existing mental health problems and trauma histories and members of the LGBTQIA+ community, as well as women who may be exposed to gender-based experiences of family violence and employment instability. The latter should be considered in relation to prior studies that have indicated that gender-based violence may increase in the context of major disasters,^36^ as such disasters can create new vulnerabilities and opportunities for men to use violence and controlling behaviours (for example, owing to insecure financial circumstances and temporary housing arrangements following the disaster).^37^ Relevant studies have also shown that Australian women (relative to men) experienced increased rates of job loss and unpaid work during the initial years of the pandemic;^38^ this signals the importance of adopting gendered perspectives on disaster and pandemic impacts that recognise the intersecting forms of adversity (including precarious economic circumstances) faced by many women in disaster-affected communities.

### Limitations

The data for this project were collected around 2 years after the Black Summer bushfires and early cases of COVID-19 in Australia, and accordingly they provide a snapshot of experiences at this point in time. Data collection was limited to three rural areas in Australia (NSW, Victoria and South Australia), and findings may not be generalisable to other jurisdictions. Participants were self-selected, and it is plausible that community members and help providers who were significantly adversely affected, while being least marginalised, were more likely to choose to participate. Finally, the sample was composed predominantly of women and so the experiences of other genders were not well represented.

### Implications

The findings of this study portray a series of impacts of overlapping events and thus situate the consequences of the pandemic in relation to parallel processes of adjustment and recovery from bushfires. They also highlight impacts and expectations of additional disasters that may further complicate recovery for some communities and thus identify the need for long-term and integrated approaches to resourcing and governing disaster resilience and recovery processes. At the broadest level, this may require the establishment of permanent agencies with responsibilities for enabling community preparedness, resilience and recovery from increasing numbers of climate-induced disasters and coordinating strategies to address events that have overlapping and long-term impacts that also extend across jurisdictions (including state and territory boundaries in Australia). Such strategies are likely to require long-term funding programmes that are simplified and streamlined (and can be accessed for any disaster) and are also trauma-informed. The current findings suggest that such funding should focus in part on the development and/or protection of ‘social infrastructure’ (i.e. spaces and places that create and maintain social connection), which may be critical for developing stocks of social capital in disaster settings.^39^ There should also be investment in strategies that can leverage such social infrastructure in order to maximise well-being outcomes for individuals and may be informed by emerging literature on the positive and negative health implications of group membership and social identification (which is referenced increasingly in terms of the ‘social cure’ approach).^40^

The findings also identified significant challenges in developing and maintaining a local workforce of help providers that can continue supporting mental health and well-being in communities affected by disasters and the pandemic. This is positioned in relation to pre-existing shortages of mental health professionals in many parts of Australia and outside metropolitan areas,^41^ which indicate the probable need for multi-component approaches to addressing workforce issues in the context of multiple disasters. These may involve the maintenance and expansion of flexible service delivery options that can help distribute and reduce demand on local providers, for example, via provision of telehealth services and online support programmes, as well as ‘fly-in-fly-out’ service providers (which, although imperfect, can all help to supplement and reduce burdens on the local workforce). They may also involve the implementation of stepped-care models of mental health service provision, which are based on hierarchies of interventions, from least to most intensive, that are also matched to individual needs. Incorporating principles of stepped care can assist by shifting some forms of support provision to trained non-specialists who provide low level interventions, thus making efficient use of the specialist workforce to support people with complex issues.

Finally, the current findings highlight many pre-existing mental health issues and forms of trauma that intersect with disaster and pandemic impacts, including gender-based experiences of family violence. These signal the need for strategies to increase support for post-traumatic mental health problems and also enhance the integration and pathways across different services. This may involve the commissioning of specialist services for post-traumatic mental health problems linked to all forms of trauma, as well as the design of programmes to enhance integration and referrals across services targeting different issues. These include services for disaster recovery, mental health, housing and family violence, which should all be expected to co-occur regularly given the increasing numbers of compounding disasters that are anticipated in the future.

## Data Availability

The data that support the findings of this study are available on request from the corresponding author, C.B. The data are not publicly available as they contain information that could compromise the privacy of research participants.
